# Quantification of the Adhesion Strength of *Candida albicans* to Tooth Enamel

**DOI:** 10.3390/microorganisms9112213

**Published:** 2021-10-25

**Authors:** Gubesh Gunaratnam, Johanna Dudek, Philipp Jung, Sören L. Becker, Karin Jacobs, Markus Bischoff, Matthias Hannig

**Affiliations:** 1 Institute of Medical Microbiology and Hygiene, Saarland University, 66421 Homburg, Germany; philipp.jung@uks.eu (P.J.); soeren.becker@uks.eu (S.L.B.); markus.bischoff@uks.eu (M.B.); 2 Clinic of Operative Dentistry and Periodontology, Saarland University, 66421 Homburg, Germany; johanna.dudek@uks.eu (J.D.); matthias.hannig@uks.eu (M.H.); 3 Experimental Physics, Saarland University, 66123 Saarbrücken, Germany; k.jacobs@physik.uni-saarland.de; 4 Max Planck School Matter to Life, 69120 Heidelberg, Germany

**Keywords:** single-cell force spectroscopy, *Candida albicans*, dental caries, adhesion

## Abstract

Caries is one of the most prevalent diseases worldwide, which is caused by the degradation of the tooth enamel surface. In earlier research the opportunistic pathogen *Candida albicans* has been associated with the formation of caries in children. Colonization of teeth by *C. albicans* starts with the initial adhesion of individual yeast cells to the tooth enamel surface. In this study, we visualized the initial colonization of *C. albicans* yeast cells on pellicle-covered enamel by scanning electron microscopy. To quantitatively unravel the initial adhesion strength, we applied fluidic force microscopy-based single-cell force spectroscopy to examine the key adhesion parameters adhesion force, rupture length and de-adhesion work. We analyzed single saliva-treated or untreated yeast cells on tooth enamel specimens with or without salivary pellicle. Under all tested conditions, adhesion forces in the lower nanonewton range were determined. Furthermore, we have found that all adhesion parameters were enhanced on the pellicle-covered compared to the uncovered enamel. Our data suggest that initial adhesion occurs through a strong interaction between yeast cell wall-associated adhesins and the salivary pellicle. Future SCFS studies may show whether specific management of the salivary pellicle reduces the adhesion of *C. albicans* on teeth and thus contributes to caries prophylaxis.

## 1. Introduction

The opportunistic pathogen *Candida albicans* is a common fungal species found in the human oral cavity. It is a polymorphic organism growing as yeast cells, or as filamentous true hyphae or pseudohyphae. *C. albicans* builds well-structured and dynamic biofilms which are the preferred growth form to ensure microbial presence in the oral cavity [1,2,3]. Multi- or dual-species biofilms formed between *C. albicans* and bacteria, such as *Actinomyces viscosus* or *Streptococcus mutans,* are often associated with denture stomatitis, periodontitis, and dental caries, especially in children with severe early childhood caries (ECC) [4,5,6].

ECC is one of the most prevalent children diseases and remains a global public health concern [7]. Particularly, teeth infiltrations with *C. albicans* were found in a high percentage of children with caries [8], and deep carious lesions with mono or dual *C. albicans* and non-*albicans Candida* species were found in a recent work [9]. Furthermore, in the dental plaques of ECC-suffering children a 3-fold higher number of *C. albicans* cells was found than in the saliva of these children [10]. It has been stated that a high oral *Candida* spp. carriage correlates with the severity of ECC [9,11,12].

In the oral cavity, saliva-exposed tooth surfaces are covered by a salivary pellicle, a tooth bio-conditioning formed by the adsorption of macromolecules from saliva [13]. Beyond protective properties, the role of salivary pellicle as a binding platform for *C. albicans* has been thoroughly investigated [14,15,16,17,18,19,20,21]. Studies reported the adhesion of *C. albicans* to both, uncovered or saliva-covered tooth enamel, or to hydroxyapatite, the mineral component of the enamel [21,22]. Others demonstrated that adsorbed saliva on hydroxyapatite clearly increased the adhesion of *C. albicans* to this substratum [23,24]. However, these studies determined the number of adherent cells in adhesion assays, which last several minutes to hours. To characterize the adhesion process of *C. albicans* to tooth enamel fully, it is necessary to know the adhesion forces or the de-adhesion work of single *C. albicans* yeast cells on untreated or saliva-treated tooth enamel, ideally at a very early (initial) contact formation-time point.

Fluidic force microscopy (FluidFM)-based single-cell force spectroscopy (SCFS) is a force-sensitive technique to study the adhesion strength of individual bacterial, fungal or mammalian cells to a substratum, such as biological or medically-relevant surfaces [25,26,27,28]. The FluidFM combines an atomic force microscope (AFM) cantilever with a microfluidic channel that can be used to immobilize single cells at the free end of the cantilever [25,27,29]. Additionally, AFM-based SCFS allows to quantify the adhesion of microbial cells covered with bodily fluids, like saliva or blood plasma [27,28,30,31].

In this study, FluidFM-based SCFS was used to quantify the initial adhesion strength between *C. albicans* yeast cells and bovine tooth enamel specimens for short contact times (0 s and 5 s). To analyze the influence of saliva on the adhesion, saliva-treated and untreated yeast cells and enamel were used. To mimic the conditions of the adhesion within the oral cavity more closely, we additionally studied the initial adhesion of the yeast cells to an in situ-formed 3 min-pellicle. Furthermore, the colonization of *C. albicans* on tooth enamel surfaces was visualized by scanning electron microscopy (SEM). Under all tested conditions, we found a strong adhesion of *C. albicans* yeast cells to tooth enamel specimens with adhesion forces in the nanonewton and de-adhesion work in the femtojoule range. For both saliva-untreated and -treated yeast cells, the adhesion strength was significantly higher on the pellicle-covered than on the uncovered tooth enamel surface, already noticeable at the indicated short contact times.

## 2. Materials and Methods

### 2.1. Yeast Strain and Cultivation

*C. albicans* (Robin) Berkhout strain ATCC 10231 was obtained from the German Collection of Microorganisms and Cell Cultures (DSMZ). The yeast cultivation protocol was adapted from a work published earlier [27]. Briefly, strain ATCC 10231 was cultivated on solid trypticase soy agar (TSA) plates with 5% sheep blood and kept for a maximum of 2 weeks at 4 °C. For the experiments, yeast-phase cells were grown in yeast extract peptone dextrose (YPD) liquid medium (Becton Dickinson GmbH, Heidelberg, Germany) for 19 h under aerobic conditions at 150 RPM and 37 °C, with a flask to medium ratio of 12.5 to 1.

### 2.2. Enamel Samples

Enamel samples (3 × 4 × 1 mm) were prepared from the vestibular surfaces of bovine incisor teeth. The surfaces were progressively polished by wet grinding with up to 4000 grit (Buehler, Düsseldorf, Germany) and purified from impurities by incubation in 3% NaOCl for 3 min, ultrasonication with distilled water for 10 min, incubation in 70% isopropyl alcohol for 15 min and final incubation in distilled water for at least 12 h. For SCFS measurement, the enamel sample was fixed on a FluoroDish Cell Culture Dish (World Precision Instruments GmbH, Friedberg, Germany) with instant glue (Pattex, Düsseldorf, Germany) and immediately covered with 20 µL PBS.

### 2.3. Human Subjects

Orally examined healthy subjects gave their informed consent to participate in this study. The study was conducted in accordance with the Declaration of Helsinki. Saliva and pellicle collection protocols were approved by the medical ethics committee of the Medical Association of Saarland, Germany (proposal # 238/03, 2016, and 54/21, 2021).

### 2.4. Saliva Treatment of Yeast and Enamel

Human saliva was collected from 12 subjects, centrifuged twice for 10 min at 14,000 rpm and 2 °C to remove the oral microflora, such as yeasts and bacteria, pooled, and stored at −80 °C until use.

For the treatment with saliva, yeast cells of the 19-h liquid culture were centrifuged at 5000 rpm for 5 min and washed twice in 1 mL PBS. Subsequently, 1 mL of a cell suspension with an OD_600_ of 3 was centrifuged, the yeast pellet was resuspended in 100 µL saliva and incubated for 15 min at 37 °C. After incubation, the cell-saliva mixture was filled up to 1.5 mL with PBS and centrifuged. The washing step was repeated once with 1 mL PBS. The pellet was then resuspended in 1 mL PBS.

For in vitro pellicle formation on tooth enamel, the polished enamel sample was fixed on a FluoroDish Cell Culture Dish, covered with 20 µL saliva for 3 min at 37 °C, washed 4 times with PBS, and covered with 20 µL PBS prior to SCFS.

### 2.5. Oral Exposure

In situ pellicle formation was performed as previously described [32]. After 3 min of oral exposure, enamel samples were removed from the oral cavity, washed with distilled water, dried with a tissue on the bottom side of the tooth enamel, fixed on FluoroDish Cell Culture Dishes and covered with 20 µL PBS.

### 2.6. Single-Cell Force Spectroscopy

For SCFS, 1 µL of a diluted (~OD_600_ of 0.3) yeast cell liquid culture (see above) was directly pipetted on the surface of the FluoroDish into 2 µL PBS in a distance of ~0.5 cm away from the tooth enamel sample. Shortly after their deposition, yeast cells were covered together with the tooth enamel in a single drop of PBS. Yeast cells were allowed to sediment for 15 min in the closed dish without drying.

SCFS experiments were performed on a Flex-Bio atomic force microscope (Nanosurf GmbH, Liestal, Switzerland) with a FluidFM^®^ module mounted on top of a Zeiss Observer Z1 microscope (Carl Zeiss, Jena, Germany). Tipless FluidFM micropipettes (Cytosurge AG, Glattbrug, Switzerland) with a nominal spring constant of 0.3 N/m and an aperture of 2 µm were used. Ovoid yeast cells were brought into optical focus (500-fold magnification). Cells with a roughly 4 µm × 5 µm size were selected for SCFS measurements. For functionalization, a single yeast cell was approached with a cantilever setpoint of 10 nN and a pressure of −400 mbar for a few hundreds of milliseconds. A pressure of −350 mbar was adjusted to hold the cell during measurements. To characterize the adhesion properties, force-distance curves on the enamel were conducted with a ramp size of 5 µm, approach and retraction speed of 800 nm/s, and a force setpoint of 10 nN. Surface contact time delays of 0 s and 5 s have been tested. Five force-distance curves were recorded for each cell, with a lateral distance of at least 10 µm between reading points to ensure the same spot was not probed twice. The retraction part of the force-distance curves was quantified for adhesion force, rupture length and de-adhesion work with SPIP software version 6.6.2 (Image Metrology, Hørsholm, Denmark) and OriginPro 2019b (OriginLab, Northampton, MA, USA).

### 2.7. Scanning Electron Microscopy (SEM)

Saliva-treated *C. albicans* yeast cells (see above) were incubated for 15 min on enamel surfaces covered with an in situ-generated 3 min-pellicle (see above), and subsequently fixed overnight at 4 °C with fixing solution (4% glutaraldehyde; 0.1 M cacodylate/HCl pH 6.8). After washing 5 times with a cacodylate buffer (0.1 M cacodylate/HCl pH 7.5) for 10 min and contrasting with 2% osmium tetroxide in the cacodylate buffer for 2 h, the samples were washed 5 times with distilled water, dehydrated in ethanol solutions with increasing concentrations (50%, 70%, 90%, 100%) and dried overnight. Before sputtering with carbon (Bal-tec SCD 030 sputter coater, Leica Microsystems, Vienna, Austria) and gold (Bal-tec SCD 005 sputter coater, Leica Microsystems, Vienna, Austria) the specimens were fractured in 2 pieces to enable viewing from the site. Imaging was performed using a Philips/FEI XL30 ESEM FEG microscope (FEI, Eindhoven, The Netherlands).

### 2.8. Statistical Analyses

The statistical significance of changes of all data distributions between 2 groups was assessed by Mann-Whitney U test and between 3 groups by Kruskal-Wallis test followed by post-hoc analysis implemented in the OriginPro2019b software (OriginLab, Northampton, MA, USA). Identified *p*-values < 0.05 were considered statistically significant.

## 3. Results

**Figure 1:**
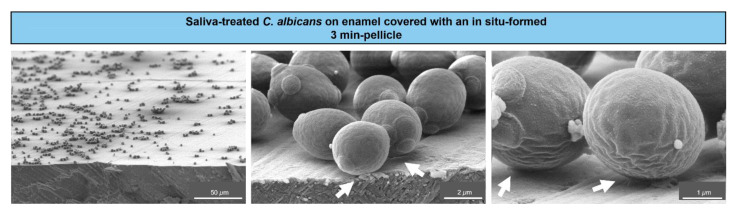
Scanning electron microscopy of *C. albicans* colonizing tooth enamel. Representative micrographs of *C. albicans* yeast cells from a liquid growth culture and after saliva treatment, which were left to adhere on tooth enamel covered with an in situ-formed 3 min-pellicle. Arrows indicate the contact sites between the cells and the salivary pellicle.

To visualize the initial adhesion of *C. albicans* yeast cells to tooth enamel, SEM was applied. The resulting micrographs showed saliva-treated yeast cells adhering to in-situ pellicle-covered enamel (Figure 1).

A distinct coverage of the enamel surface by *C. albicans* cells was visible at lower magnifications (left panel). Higher magnifications (middle and right panels) revealed close contact sites between the cells and the salivary pellicle, as indicated by the arrows. This illustrated an early settlement of individual *C. albicans* cells on tooth enamel at close to physiological intraoral conditions.

**Figure 2:**
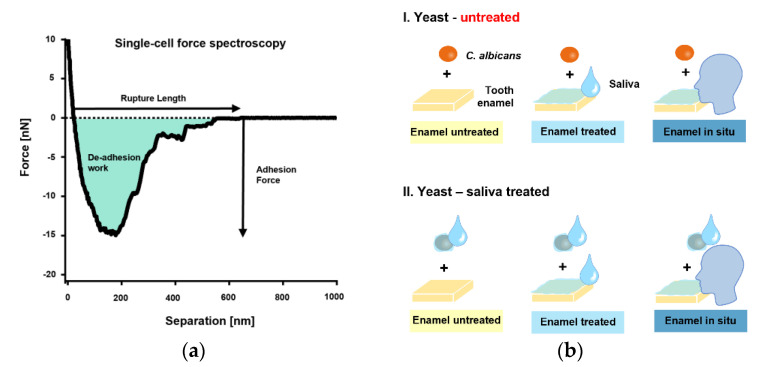
Single-cell force spectroscopy with *C. albicans* and experimental conditions. (**a**): FluidFM was used to bring a single cell into contact with a bovine tooth enamel sample for short surface contact times of 0 s or 5 s. The yeast probe was then retracted, and the adhesion force, rupture lengths and de-adhesion work were quantified (**b**): The experimental design.

The initial single-cell adhesion properties (i.e., adhesion force [F_adh_], rupture length [L_rupt_], and de-adhesion work [W_adh_]) of single *C. albicans* yeast cells on bovine tooth enamel were next quantified by SCFS (Figure 2a). An overview of the tested, different measurement conditions is shown in Figure 2b.

**Figure 3:**
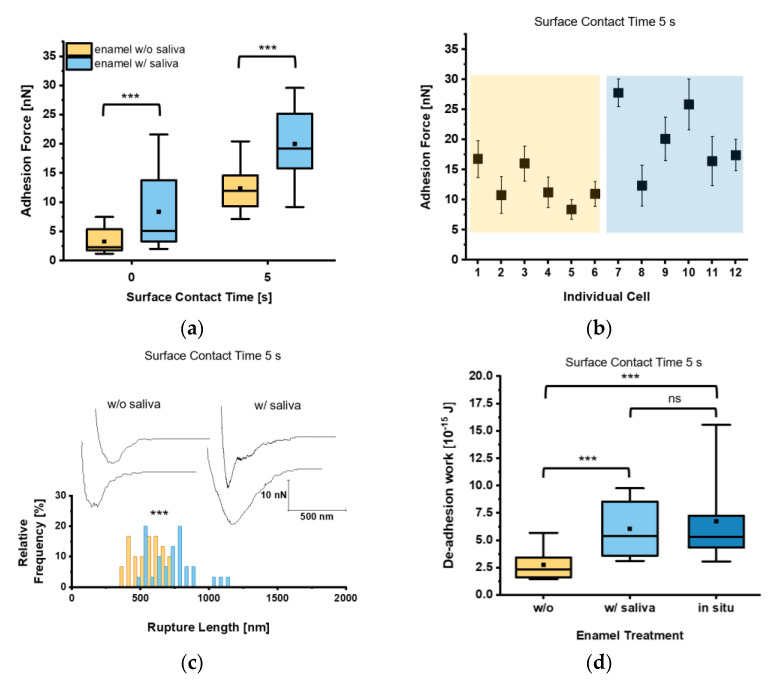
Adhesion of untreated *Candida albicans* yeast cells to tooth enamel and the impact of salivary pellicle. (**a**) Adhesion forces of *C. albicans* yeast cells to untreated or saliva-treated enamel and for surface contact time delays of 0 s and 5 s. Data are presented in box and whisker plots (min-to-max), with median (horizontal line) and mean value (closed square). For each box 30 data points of six individual cells with five data points per cell are depicted. ***, *p* < 0.001 (Mann-Whitney U Test). (**b**) Adhesion forces of individual yeast cells. Shaded areas indicate treatment of the enamel w/o (orange) or w/(blue) saliva. Mean adhesion forces and standard deviations are indicated. (**c**) Representative retraction curves for untreated (**left**) and saliva-treated (**right**) enamel and rupture lengths for a surface contact time delay of 5 s. The histogram for rupture lengths consists of a bin size equivalent to 50 nm. ***, *p* < 0.001 (Mann-Whitney U Test). (**d**) De-adhesion work of yeast cells for a contact time of 5 s for untreated, saliva-treated, and in situ-exposed tooth enamel. Data are presented in box and whisker plots (min-to-max), with median (horizontal line) and average (closed square). For each box 30 data points of six individual cells with five data points per cell are depicted. ns, not significant; ***, *p* < 0.001 (Kruskal-Wallis test followed by post-hoc analysis).

Firstly, the adhesion force F_adh_ of single *C. albicans* yeast cells on bovine tooth enamel was determined with or without the presence of human saliva. When we looked at the mean adhesion forces for a short contact time (t = 0 s), F_adh_ of 3.3 ± 2.1 nN and 8.4 ± 6.2 nN were determined on untreated and human saliva-treated tooth enamel, respectively. To test F_adh_ for a higher contact time, we increased the surface contact time delay to t = 5 s, which resulted in an elevated F_adh_ of 12.4 ± 3.6 nN and 20.0 ± 6.0 nN for the respective tooth enamel conditions (Figure 3a).

These results indicate that salivary macromolecules bound to the tooth enamel surface facilitate the adhesion of the yeast towards the surface of the tooth. Furthermore, expanding the surface contact time delay from 0 s to 5 s increases F_adh_ in general. At a closer look on the individual cell adhesion forces, we frequently observed high F_adh_ on saliva-treated enamel and lower F_adh_ on untreated tooth enamel (Figure 3b). Analyzing L_rupt_ as well, which marks the distance where the last bonds between *C. albicans* and the tooth enamel are disrupted, this parameter was significantly higher on saliva-treated tooth enamel (720.9 ± 167.3 nm) than on untreated tooth enamel (558.8 ± 111.2 nm; Figure 3c). This strongly indicates an interaction between *C. albicans* cell-surface components and saliva biomolecules on the enamel surface. Investigation of the de-adhesion work W_adh_, which comprises all binding and rupture events included in the detachment process of the yeast cell from the surface, yielded in a mean W_adh_ significantly increased by a factor of 2.2 on saliva-treated enamel when compared to untreated enamel for a surface contact time delay of 5 s (Figure 3d). Furthermore, W_adh_ of *C. albicans* yeast cells on an in situ-generated 3 min pellicle on tooth enamel was also determined. Here, we found W_adh_ values similar to those recorded on saliva-treated enamel, which were by a factor of 2.5 higher than on untreated tooth enamel (Figure 3d).

**Figure 4:**
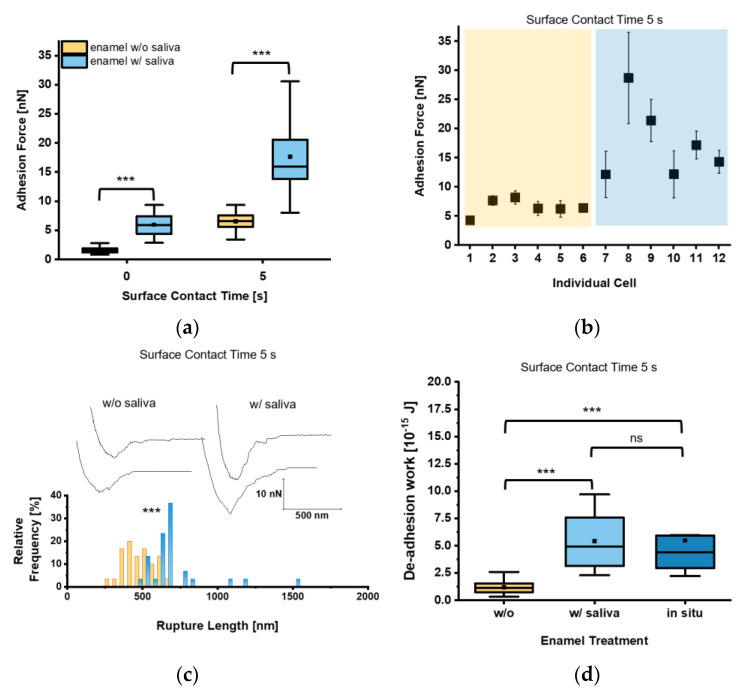
Adhesion of saliva-treated *Candida albicans* yeast cells to tooth enamel. (**a**) Adhesion forces of saliva-treated *C. albicans* yeast cells to untreated or saliva-treated enamel and for surface contact time delays of 0 s and 5 s. Data are presented in box and whisker plots (min-to-max), with median (horizontal line) and mean value (closed square). For each box 30 data points of six individual cells with five data points per cells are depicted. ***, *p* < 0.001 (Mann-Whitney U Test). (**b**) Adhesion forces of individual yeast cells. Shaded areas indicate treatment of the enamel w/o (orange) or w/(blue) saliva. Mean adhesion forces and standard deviations are indicated. (**c**) Representative retraction curves for untreated (left) and saliva-treated (right) enamel and rupture lengths for a surface contact time delay of 5 s are shown. The histogram for rupture lengths consists of a bin size equivalent to 50 nm. ***, *p* < 0.001 (Mann-Whitney U Test). (**d**) De-adhesion work of yeast cells for a surface contact time delay of 5 s for the untreated, saliva-treated, and in situ-exposed tooth enamel. Data from five to six individual cells with five data points per cell are presented in box and whisker plots (min-to-max), with median (horizontal line) and average (closed square). ns, not significant; ***, *p* < 0.001 (Kruskal-Wallis test followed by post-hoc analysis).

Given that circulating *C. albicans* yeast cells are exposed to saliva in the oral cavity, we next investigated whether and how the decoration of the yeast cell surface with salivary factors affected the adhesion of *C. albicans* to tooth enamel. Our measurements revealed for t = 0 s F_adh_ values of 1.6 ± 0.5 nN and 6.0 ± 2.0 nN for saliva-treated yeast cells on untreated and saliva-treated tooth enamel, respectively (Figure 4a). Increasing the surface contact time delay to 5 s resulted in an increase of the F_adh_ to 6.5 ± 1.5 nN on untreated enamel and to 17.7 ± 6.8 nN on saliva-treated enamel. Here, all individual cells brought into contact with the saliva-treated enamel bound with strong F_adh_, while on untreated tooth enamel F_adh_ was comparably low in all cases (Figure 4b). Values for L_rupt_ reached 703.2 ± 215.9 nm on saliva-treated tooth enamel and were significantly higher than on untreated tooth enamel (479.4 ± 105.1 nm), indicating an extended binding of macro-molecules of the saliva-treated yeast cell surface to salivary macromolecules deposited on the enamel (Figure 4c). W_adh_ of the saliva-treated yeast cells was also strongly increased on saliva-treated tooth enamel (Figure 4d), and more than 4-fold higher when compared to the untreated equivalent. In order to further approach the putative adhesion scenario encountered between *C. albicans* and the enamel in the oral cavity, we also determined W_adh_ produced by saliva-treated yeast cells on in situ-generated pellicles (Figure 4d). The W_adh_ values observed here were in a similar range to those observed for saliva-treated enamel and confirmed the strong adhesion strength of the saliva-treated yeast cells on salivary pellicles (Figure 4d).

**Figure 5:**
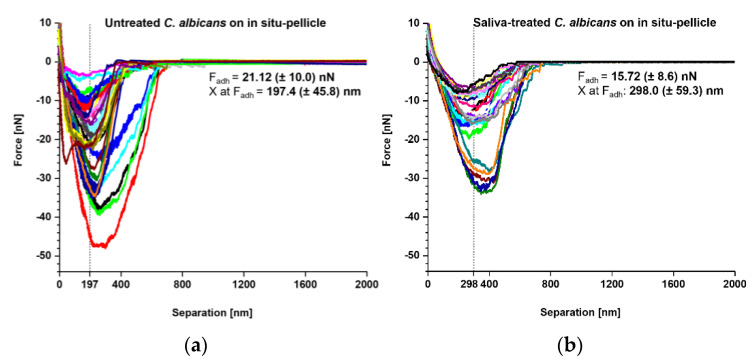
Comparison of adhesion forces between untreated and saliva-treated *Candida albicans* yeast cells on in situ pellicles of tooth enamel. Overlay of retraction curves on in situ-formed pellicles on tooth enamel. (**a**) Data for untreated *C. albicans* yeast cells (*n* = 35) and (**b**) for saliva-treated *C. albicans* yeast cells (*n* = 25). In each panel the mean adhesion force (F_adh_) and the mean separation length of the adhesion force (X at F_adh_) are given.

Our SCFS experiments demonstrated that both naïve and saliva-treated *C. albicans* yeast cells bound strongly to tooth enamel that was covered with a salivary pellicle. Thus, we wondered whether the underlying adhesion mechanism might be the same. To examine this, we produced an overlay of retraction curves generated with naïve and saliva-treated *C. albicans* yeast cells on in situ-formed pellicles to examine the adhesion mechanism under preferably physiological conditions (Figure 5). Here, we observed F_adh_ values of 21.12 ± 10.0 nN for untreated yeast cells and 15.72 ± 8.6 nN for saliva-treated yeast cells (Figure 5a,b insets). Interestingly, for untreated yeast cells, the F_adh_ maxima were observed at a separation distance of 197.4 ± 45.8 nm, while for saliva-treated yeast cells the separation distances were shifted to 298.0 ± 59.3 nm. These findings demonstrate that the salivary treatment of the *C. albicans* yeast-cell surface moves the separation of F_adh_ by approximately 100 nm. Possible explanations for this observation might be that the binding of some cell-wall components at shorter distances was inhibited, and/or that the binding mechanism for some of the yeast adhesins was altered or masked in the presence of soluble salivary biomolecules.

## 4. Discussion

In this work, the initial binding of *C. albicans* to in-situ pellicles on enamel was visualized by scanning electron microscopy, showing adherent yeast cells on the tooth enamel surface with close contact sites to the salivary pellicle. Earlier observations of a 10-days mature *C. albicans* biofilm on cleaned tooth enamel demonstrated a complex conglomerate of yeast cells and hyphae, and mycelium formation [33]. Thus, the initial binding of single *C. albicans* yeast cells can lead to the colonization of the tooth surface, and might serve as a bridging factor for other microorganisms to build up polymicrobial, cariogenic biofilms [9,33,34,35].

Our FluidFM-based SCFS studies demonstrated adhesion forces for *C. albicans* yeast cells on tooth enamel in the lower nanonewton range, which is in the same dimension as for this yeast morphotype on artificial surfaces, such as hydrophobic and hydrophilic dodecyl phosphate surfaces, nanostructured gold surfaces, or polyurethane-based central venous catheter (CVC) tubing [25,27,36]. Additionally, similar to the findings made with *C. albicans*-germinated cells on human blood plasma-coated CVC tubing [27], we observed enhanced adhesion for *C. albicans* yeast cells on the bio-conditioned, pellicle-covered tooth enamel. One putative explanation for this observation is a pronounced tethering of yeast cell-surface adhesins to the salivary components in the pellicle. The outer cell wall of *C. albicans* consists among others of mannoproteins with adhesive functions [37]. Such adhesion factors were found to be crucial in *Staphylococcus aureus* for the adhesion on silicon wafers, due to strong tethering and stretching of proteins, and the stepwise unlocking of protein domains [30,38]. We assume that the same is true for *C. albicans* yeast cells displaying stretchable mannoproteins on their surface [39], such as the Als proteins, which exhibit adhesive functions and are ideal candidates for abiotic surface binding or interactions with human extracellular proteins [40,41]. Indeed, recent work reported that the yeast proteins Als1, endoglucanase Bgl2, and the cell wall-anchored Ecm33 contribute to the binding of yeast cells to saliva-covered hydroxyapatite, the mineral component of enamel [24]. Earlier work demonstrated already that *C. albicans* yeast cells adhere in larger quantities to saliva-treated HAP surfaces than to untreated HAP [23,24]. Potential ligands in the salivary pellicle might be basic proline-rich proteins (PRPs), such as IB–6, the calcium-binding protein statherin, and BPIFA 2 [32,42,43]. Interestingly, the adhesion forces, rupture lengths and the de-adhesion work show a larger range of values on saliva-treated enamel than on untreated enamel. We assume that this is due to an individual cell-wall composition of yeast adhesins that specifically bind salivary ligands, and because of an inhomogeneous deposition of salivary biomolecules on the substratum [44]. The adhesion of the yeast cell to the untreated tooth enamel is mediated through unspecific interactions with a narrow range for adhesion forces. However, the presence of pellicle-specific interactions between yeast adhesins and individual salivary ligands might lead to weaker or stronger binding forces, depending on the number of yeast adhesins coming into contact with the pellicle and on the type and number of ligands available in the pellicle [37,42]. Such a scenario is also in line with the increase in rupture lengths, when loosely adsorbed salivary macromolecules that come into contact with the yeast cell surface will be stretched in the adhesion process.

It is worth noticing that the supportive effect of pellicle for the initial adhesion of *C. albicans* to tooth enamel might be seen only with early pellicles (i.e., 3 min-pellicle), as previous work observed a reduced adhesion capacity of *C. albicans* to dental acrylic surfaces decorated with matured salivary pellicles (i.e., 18 h-pellicle) compared to the PBS control [16]. However, direct comparison between SCFS and traditional, cell number determining-adhesion assays should be made with caution. SCFS is a force-sensitive method and directly quantifies the initial adhesion of a cell after contact to a substratum. Future SCFS studies should thus investigate the adhesion of *C. albicans* to matured pellicles, and include *C. albicans* deletion mutants that do not synthesize single or multiple adhesion factors. This will help to further elucidate the molecular mechanisms behind the adhesion of *C. albicans* to pellicle-covered enamel.

Given that *C. albicans* cells circulating in the oral cavity are constantly exposed to saliva, we also investigated whether and how the covering of the yeast cell surface with saliva affects the cell’s ability to adhere to the enamel. Again, we observed a stronger *C. albicans* adhesion strength on pellicle-covered than on untreated enamel. This suggests that the binding of *C. albicans* and its macromolecular adhesins to available salivary ligands in the pellicles is largely preserved. One explanation for this might be the maintenance of the binding activity of the fungal adhesins through their capacity to differentiate between salivary biomolecules in solution and in the pellicle. It has been suggested that *C. albicans* does not bind some salivary ligands (e.g., PRPs), when these are in solution, which has been shown for the oral bacterium *Streptococcus gordonii* [42,45]. It cannot be excluded, however, that the newly yeast cell surface-attached salivary components might mediate the adhesion to the pellicle as well, as has been suggested for saliva-coated *S. aureus* bacterial cells on saliva-treated titanium [31].

In our SCFS studies, we noticed a prolonged mean separation distance for the adhesion force maxima for saliva-treated yeast cells probed on in situ pellicles when compared to untreated yeast cells probed on the same kind of surface. The most likely explanation for this observation is that salivary biomolecules, which were adsorbed on the yeast cell surface, interfere with the binding of the *C. albicans* adhesins to the pellicle components. This scenario is similar to findings made with *C. albicans* and fibronectin, in which a reduced binding of *C. albicans* to immobilized fibronectin was observed when the yeast cells were pre-exposed to fibronectin in solution [46]. The observed shift of the adhesion force by a separation of almost 100 nm indicates that small-sized adhesins, which probably contribute substantially to the strong adhesion force observed for untreated yeast cells, are hindered from binding in saliva-treated yeast cells to the same type of surface by the interference of salivary components. In contrast, the remaining free yeast adhesins are not prevented from tethering and contribute to the observed larger separation distances for the adhesion force.

Although our SCFS studies do not provide any physicochemical information on the nature behind the adhesion strength of *C. albicans* to tooth enamel with or without pellicle, our force-distance curves indicate a simultaneous stretching of adhesive bonds, predominantly occurring between *C. albicans* and dental pellicles. It is likely that the nano roughness of the surface plays a key role here during these adhesion events. We have previously shown that an in situ-formed 10 min-pellicle yielded in globular agglomerates in nm dimension on the enamel [47]. From this, it is tempting to speculate that a synergistic effect between surface nano-roughness and specific interactions of the involved binding macromolecules contributes to the stretching of the adhesive bonds. Support for this hypothesis is given by a recent study showing that a coating of glass with gold particles with a density of 61 nanoparticles per µm^2^ was sufficient to significantly enhance the adhesion forces of *C. albicans* to the gold particles-treated surface when compared to the naïve glass surface [36]. Notably, the retraction curves for *C. albicans* yeast cells brought into contact with the gold-covered glass surface were reminiscent to our retraction curves observed with *C. albicans* on pellicle-covered enamel and suggest that an increase in surface nano-roughness might be already sufficient to increase the adhesion force, while specific receptor-ligand interactions may further intensify this interaction [40]. Moreover, theoretical models can be refined with our results, revealing binding processes, types of intermolecular forces and the spatial distribution of adhesion sites [48,49].

## 5. Conclusions

Our SCFS studies demonstrate that *C. albicans* yeast cells adhere strongly with nN adhesion forces and fJ de-adhesion work to tooth enamel, which suggests that this opportunistic pathogen possesses a repertoire of virulence factors to colonize the tooth surface. Especially on salivary pellicle, which is formed on the enamel within seconds after tooth brushing, *C. albicans* utilizes adsorbed ligands to bind to the tooth, which probably supports the microbe to withstand salivary flow and being swallowed by the host. Future experimental and theoretical studies will show whether the specific management of salivary pellicles leads to a reduced adhesion of *C. albicans* and can thus contribute to caries prophylaxis.

## Data Availability

The datasets generated and analyzed during the current study are available from the corresponding author on reasonable request.
